# Current Awareness on Comparative and Functional Genomics

**DOI:** 10.1002/(SICI)1097-0061(200004)17:1<71::AID-YEA7>3.0.CO;2-K

**Published:** 2000


**Yeast**
*Yeast* 2000; **17:** 71–78

### Current awareness on comparative and functional genomics

### 1 Reviews & symposia

Alda M. 1999. Dalhousie Univ, Dept Psychiat, Abbie J Lane Bldg, 5909 Jubilee Rd, Halifax, Nova Scotia, Canada B3H 2E2. Pharmacogenetics of lithium response in bipolar disorder (Review). *J Psychiatry Neurosci* **24:** (2) 154.

Bassett DE, Eisen MB, Boguski MS. 1999. Rosetta Inpharmat, Kirkland, Wa 98034, USA. Gene expression informatics: It's all in your mine (Review). *Nat Genet* **21:** (Suppl) 51.

Bowtell DDL. 1999. Peter MacCallum Canc Inst, Locked Bag 1, A'Beckett St, Melbourne, Vic 3000, Australia. Options available: From start to finish - for obtaining expression data by microarray (Review). *Nat Genet* **21:** (Suppl) 25.

Brown PO, Botstein D. 1999. Stanford Univ, Sch Med, Dept Biochem, Stanford, Ca 94305, USA. Exploring the new world of the genome with DNA microarrays (Review). *Nat Genet* **21:** (Suppl) 33.

Chakravarti A. 1999. Case Western Reserve Univ, Sch Med, Dept Genet, Cleveland, Oh 44106, USA. Population genetics: Making sense out of sequence. *Nat Genet* **21:** (Suppl) 56.

Cheung VG, Morley M, Aguilar F, Massimi A, Kucherlapati R, Childs G. 1999. Univ Penn, Dept Pediat, Philadelphia, Pa 19104, USA. Making and reading microarrays (Review). *Nat Genet* **21:** (Suppl) 15.

Cole KA, Krizman DB, Emmert-Buck MR. 1999. NIH/NCI, Pathol Lab, Pathogenet Unit, Bldg 10, Bethesda, Md 20892, USA. The genetics of cancer-a 3D model (Review). *Nat Genet* **21:** (Suppl) 38.

Debouck C, Goodfellow PN. 1999. SmithKline Beecham Pharmaceut, 709 Swedeland Rd, King of Prussia, Pa 19406, USA. DNA microarrays in drug discovery and development (Review). *Nat Genet* **21:** (Suppl) 48.

Duggan DJ, Bittner M, Chen YD, Meltzer P, Trent JM. 1999. NIH/NHGRI, Canc Genet Branch, Bethesda, Md 20892, USA. Expression profiling using cDNA microarrays (Review). *Nat Genet* **21:** (Suppl) 10.

Evans WE, Relling MV. 1999. St Jude Children's Hosp, Dept Pharmaceut Sci, 332 Nth Lauderdale St, Memphis, Tn 38105, USA. Pharmacogenetics: Translating functional genomics into rational therapeutics (Review). *Science* **286:** (5439) 458.

Hacia JG. 1999. NIH/NHGRI, Genet & Mol Biol Branch, Bldg 49-3A14, Bethesda, Md 20892, USA. Resequencing and mutational analysis using oligonucleotide microarrays (Review). *Nat Genet* **21:** (Suppl) 42.

Hacia JG, Collins FS*. 1999. *NIH/NHGRI, Genet & Mol Biol Branch, Bldg 49-3A14, Bethesda, Md 20892, USA. Mutational analysis using oligonucleotide microarrays (Review). *J Med Genet* **36:** (10) 730.

Henke L, Krull UJ. 1999. Univ Toronto, Dept Chem, Chem Sensors Grp, 3359 Mississauga Rd Nth, Mississauga, Ontario, Canada L5L 1C6. Immobilization technologies used for nucleic acid biosensors: A review. *Can J Anal Sci Spectrosc* **44:** (2) 61.

Hopwood DA. 1999. John Innes Inst, Ctr Plant Sci Res, Norwich Res Park, Norwich NR4 7UH, England. Forty years of genetics with *Streptomyces*: From *in vivo* through *in vitro* to *in silico*. *Microbiology* **145:** (9) 2183.

Khan J, Saal LH, Bittner ML, Chen YD, Trent JM, Meltzer PS*. 1999. *NIH, Natl Human Genome Res Inst, Canc Genet Branch, Bldg 49, Room 4A10, 49 Convent Dr, Bethesda, Md 20892, USA. Expression profiling in cancer using cDNA microarrays: Review. *Electrophoresis* **20:** (2) 223.

Khan J, Bittner ML, Chen YD, Meltzer PS, Trent JM*. 1999. *NIH, Natl Human Genome Res Inst, Canc Genet Branch, Bethesda, Md 20892, USA. DNA microarray technology: The anticipated impact on the study of human disease (Mini-Review). *Biochim Biophys Acta* **1423:** (2) M17.

Krynetski EY, Evans WE*. 1999. *St Jude Children's Hosp, 332 Nth Lauderdale, Memphis, Tn 38101, USA. Pharmacogenetics as a molecular basis for individualized drug therapy: The thiopurine *S*-methyltransferase paradigm (Review). *Pharmaceut Res* **16:** (3) 342.

Lipshutz RJ, Fodor SPA, Gingeras TR, Lockhart DJ. 1999. Affymetrix Inc, 3380 Cent Expressway, Santa Clara, Ca 95051, USA. High density synthetic oligonucleotide arrays (Review). *Nat Genet* **21:** (Suppl) 20.

Lottspeich F. 1999. Max Planck Inst Biochem, DE-82152 Martinsried, Germany. Proteome analysis: A pathway to the functional analysis of proteins (Review). *Angew Chem Int Ed* **38:** (17) 2477.

O'Brien SJ, Menotti-Raymond M, Murphy WJ, Nash WG, Wienberg J, Stanyon R, Copeland NG, Jenkins NA, Womack JE, Graves JAM. 1999. NCI, Lab Genome Diversity, Frederick, Md 21702, USA. The promise of comparative genomics in mammals (Review). *Science* **286:** (5439) 458.

Poirier J. 1999. McGill Ctr Studies Aging, 6825 Lasalle Blvd, Verdun, Quebec, Canada H4H 1R3. Apolipoprotein E4, cholinergic integrity and the pharmacogenetics of Alzheimer's disease (Review). *J Psychiatry Neurosci* **24:** (2) 147.

Shinohara A, Ogawa T. 1999. Univ Chicago, Dept Radiat & Cellular Oncol, Chicago, Il 60637, USA. Rad51/RecA protein families and the associated proteins in eukaryotes (Minireview). *Mutat Res* **435:** (1) 13.

Southern E, Mir K, Shchepinov M. 1999. Univ Oxford, Dept Biochem, Sth Parks Rd, Oxford OX1 3QU, England. Molecular interactions on microarrays (Review). *Nat Genet* **21:** (Suppl) 5.

Syvanen AC. 1999. Univ Uppsala, Acad Hosp, Ing 70, 3 Tr, Favd 2, SE-75185 Uppsala, Sweden. From gels to chips: "Minisequencing" primer extension for analysis of point mutations and single nucleotide polymorphisms (Review). *Hum Mutat* **13:** (1) 1.

Wilson DS, Szostak JW. 1999. MGH, Howard Hughes Med Inst, Boston, Ma 02114, USA. *In vitro* selection of functional nucleic acids (Review). *Annu Rev Biochem* **68:** 611.

In order to keep subscribers up-to-date with the latest developments in their field, this current awareness service is provided by John Wiley & Sons and contains newly-published material on comparative and functional genomics. Each bibliography is divided into 16 sections. 1 Reviews & symposia; 2 General; 3 Large-scale sequencing and mapping; 4 Genome evolution; 5 Comparative genomics; 6 Gene families and regulons; 7 Pharmacogenomics; 8 Large-scale mutagenesis programmes; 9 Functional complementation; 10 Transcriptomics; 11 Proteomics; 12 Protein structural genomics; 13 Metabolomics; 14 Genomic approaches to development; 15 Technological advances; 16 Bioinformatics. Within each section, articles are listed in alphabetical order with respect to author. If, in the preceding period, no publications are located relevant to any one of these headings, that section will be omitted.

Copyright © 2000 John Wiley & Sons, Ltd.

Zweiger G. 1999. Incyte Pharm, 3174 Porter Dr, Palo Alto, Ca 94304, USA. Knowledge discovery in gene-expression-microarray data: Mining the information output of the genome. *Trends Biotechnol* **17:** (11) 429.

### 2 General

Blanchard AP, Friend SH. 1999. Rosetta Inpharmat, Kirkland, Wa 98034, USA. Cheap DNA arrays: It's not all smoke and mirrors (News). *Nat Biotechnol* **17:** (10) 953.

Forrest KA. 1999. Address not available. The first hope for the "big picture" of infection: DNA microarrays (Letter). *Fertil Steril* **72:** (1) 185.

Hodgson J. 1999. Address not available. Curagen lays down markers, 120,000 of them (News). *Nat Biotechnol* **17:** (10) 951.

Hoyle B. 1999. Address not available. Giga-speed bioinformatics to power Genome Canada (News). *Nat Biotechnol* **17:** (10) 950.

Jasny BR, Hines PJ. 1999. Address not available, Genome prospecting (Introduction). *Science* **286:** (5439) 443.

Kurian KM, Watson CJ, Wyllie AH. 1999. Western Gen Hosp, Mol Med Ctr, Sir Alistair Currie CRC Labs, 3rd Floor, Crewe Rd, Edinburgh EH4 2XU, Scotland. DNA chip technology (Editorial). *J Pathol* **187:** (3) 267.

Mann M. 1999. University Sthn Denmark, Prot Interact Lab, Odense, Denmark. Quantitative proteomics? (News). *Nat Biotechnol* **17:** (10) 954.

Marshall E. 1999. Address not available. Do-it-yourself gene watching (News). *Science* **286:** (5439) 444.

Marshall E. 1999. Address not available. An array of uses: Expression patterns in strawberries, Ebola, TB and mouse cells (News). *Science* **286:** (5439) 445.

Martienssen R, Irish V. 1999. Cold Spring Harbor Lab, POB 100, Cold Spring Harbor, NY 11724, USA. Copying out our ABCs: The role of gene redundancy in interpreting genetic hierarchies (Comment). *Trends Genet* **15:** (11) 435.

McDonough PG. 1999. Address not available. The first hope for the "big picture" of infection: DNA microarrays (Editorial). *Fertil Steril* **72:** (1) 187.

Moomjy M, Cholst I, Mangieri R, Rosenwaks Z. 1999. Ctr Reprod Med & Infertil, New York, NY, USA. The first hope for the "big picture" of infection: DNA microarrays (Reply). *Fertil Steril* **72:** (1) 186.

Ouzounis C. 1999. EMBL Cambridge Outstn, European Bioinformat Inst, Res Programme, Comput Genomic Grp, Cambridge CB10 1SD, England. Orthology: Another terminology muddle (Letter). *Trends Genet* **15:** (11) 445.

Owens K, King MC. 1999. Univ Washington, Dept Genet, Box 357720, Seattle, Wa 98195, USA. Genomic views of human history (Viewpoint). *Science* **286:** (5439) 451.

Pennisi E. 1999. Address not available. Keeping genome databases clean and up to date (News). *Science* **286:** (5439) 447.

Stark GR, Gudkov AV. 1999. Cleveland Clin Fdn, Lerner Res Inst, 9500 Euclid Ave, Cleveland, Oh 44195, USA. Forward genetics in mammalian cells: Functional approaches to gene discovery. *Hum Mol Genet* **8:** (10) 1925.

Strausberg RL, Feingold EA, Klausner RD, Collins FS*. 1999. *NIH/NCI, Bethesda, Md 20892, USA. The mammalian gene collection (Viewpoint). *Science* **286:** (5439) 455.

### 3 Large-scale sequencing and mapping

Dunham I *et al.* 1999. Sanger Ctr, Wellcome Trust, Genome Campus, Cambridge CB10 1SA, England. The DNA sequence of human chromosome 22. *Nature* **402:** (6761) 489.

Lin XY, Kaul SS, Rounsley S, Shea TP, Benito MI, Town CD, Fujii CY, Mason T, Bowman CL, Barnstead M *et al*. 1999. c/o Venter JC, Celera Genomics Corp, 45 West Circle Dr, Rockville, Md 20850, USA. Sequence and analysis of chromosome 2 of the plant *Arabidopsis thaliana*. *Nature* **402:** (6763) 761.

Mayer K, Schuller C, Wambutt R, Murphy G, Volckaert, Pohl T, Dusterhoft A, Stiekema W, Entian KD, Terryn N *et al*. 1999. c/o Bevan M, John Innes Ctr Plant Sci Res, Colney Lane, Norwich NR4 7UH, England. Sequence and analysis of chromosome 4 of the plant *Arabidoposis thaliana*. *Nature* **402:** (6763) 769.

### 4 Genome evolution

Cavalier-Smith T, Beaton MJ. 1999. Univ British Columbia, Dept Bot, Canadian Inst Adv Res, Evolut Biol Program, Vancouver, Brit Columbia, Canada V6T 1Z4. The skeletal function of non-genic nuclear DNA: New evidence from ancient cell chimaeras. *Genetica* **106:** (1-2) 3.

Cebolla A, Vinardell JM, Kiss E, Olah B, Roudier F, Kondorosi A, Kondorosi E*. 1999. *CNRS, Inst Sci Vegetales, Ave Terrasse, FR-91198 Gif sur Yvette, France. The mitotic inhibitor *ccs52* is required for endoreduplication and ploidy-dependent cell enlargement in plants. *EMBO J* **18:** (16) 4476.

Costello AM, Lidstrom ME. 1999. Syracuse Univ, Dept Civil & Environm Engn, 220 Hinds Hall, Syracuse, NY 13244, USA. Molecular characterization of functional and phylogenetic genes from natural populations of methanotrophs in lake sediments. *Appl Environ Microbiol* **65:** (11) 5066.

Ferea TL, Botstein D, Brown PO*, Rosenzweig RF. 1999. *Stanford Univ, Sch Med, Dept Biochem, Howard Hughes Med Inst, Stanford, Ca 94305, USA. Systematic changes in gene expression patterns following adaptive evolution in yeast. *Proc Natl Acad Sci U S A* **96:** (17) 9721.

Granjeaud S, Naquet P, Galland F*. 1999. *Ctr Immunol Luminy, INSERM, CNRS, TAGC, Parc Sci de Luminy, Case 906, FR-13288 Marseille 09, France. An ESTs description of the new Vanin gene family conserved from fly to human. *Immunogenetics* **49:** (11-12) 964.

Hurst LD, Brunton CFA, Smith NGC. 1999. Univ Bath, Dept Biol & Biochem, Bath BA2 7AY, England. Small introns tend to occur in GC-rich regions in some but not all vertebrates. *Trends Genet* **15:** (11) 437.

Polimeno L, Lisowsky T, Francavilla A*. 1999. *Univ Bari, Cattedra Gastroenterol, Dept Gastroenterol, Viale Ennio, IT-70124 Bari, Italy. From yeast to man: From mitochondria to liver regeneration - a new essential gene family. *Ital J Gastroenterol Hepatol* **31:** (6) 494.

Santos MAS, Cheesman C, Costa V, Moradas-Ferreira P, Tuite MF. 1999. Univ Kent, Res Sch Biosci, Canterbury CT2 7NJ, England. Selective advantages created by codon ambiguity allowed for the evolution of an alternative genetic code in *Candida* spp. *Mol Microbiol* **31:** (3) 937.

Vajo Z, King LM, Jonassen T, Wilkin DJ, Ho N, Munnich A, Clarke CF, Francomano CA*. 1999. *VA Med Ctr, Dept Endocrinol & Med, 650 East Indian Sch Rd, Phoenix, Az 85012, USA. Conservation of the

Copyright © 2000 John Wiley & Sons, Ltd.

*Caenorhabditis elegans* timing gene *clk-1* from yeast to human: A gene required for ubiquinone biosynthesis with potential implications for aging. *Mamm Genome* **10:** (10) 1000.

Wierinckx A, Mercier D, Oulmouden A, Petit JM, Julien R*. 1999. *Univ Limoges, Fac Sci, Inst Biotechnol, INRA, Unite Genet Mol Anim, 123 Ave Albert Thomas, FR-87060 Limoges, France. Complete genomic organization of *futb* encoding a bovine $\alpha$3-fucosyltransferase: Exons in human orthologous genes emerged from ancestral intronic sequences. *Mol Biol Evol* **16:** (11) 1535.

### 5 Comparative genomics

Clemens S, Kim EJ, Neumann D, Schroeder JI*. 1999. *Univ Calif San Diego, Dept Biol, 9500 Gilman Dr, La Jolla, Ca 92093, USA. Tolerance to toxic metals by a gene family of phytochelatin synthases from plants and yeast. *EMBO J* **18:** (12) 3325.

Devos KM, Beales J, Nagamura Y, Sasaki T. 1999. John Innes Ctr Plant Sci Res, Norwich Res Pk, Norwich NR4 7UH, England. *Arabidopsis* - rice: Will colinearity allow gene prediction across the eudicot-monocot divide? *Genome Res* **9:** (9) 825.

Gilbert DJ, Engel H, Wang XL, Grzeschik KH, Copeland NG, Jenkins NA, Kilimann MW*. 1999. *Ruhr Univ, Inst Physiol Chem, DE-44780 Bochum, Germany. The neurobeachin gene (*Nbea*) identifies a new region of homology between mouse central chromosome 3 and human chromosome 13q13. *Mamm Genome* **10:** (10) 1030.

Hughes DC, Barratt CLR. 1999. Birmingham Women's Hosp, Reprod Biol & Genet Grp, Assisted Concept Unit, Birmingham B15 2TG, England. Identification of the true human orthologue of the mouse *Zp1* gene: Evidence for greater complexity in the mammalian zona pellucida? *Biochim Biophys Acta* **1447:** (2-3) 303.

Jareborg N, Birney E, Durbin R. 1999. Karolinska Inst, Ctr Genomics Res, SE-17177 Stockholm, Sweden. Comparative analysis of noncoding regions of 77 orthologous mouse and human gene pairs. *Genome Res* **9:** (9) 815.

Ling V, Wu PW, Finnerty HF, Sharpe AH, Gray GS, Collins M. 1999. Genet Inst Inc, Dept Immunol, 87 Cambridge Pk Rd, Cambridge, Ma 02140, USA. Complete sequence determination of the mouse and human CTLA4 gene loci: Cross-species DNA sequence similarity beyond exon borders. *Genomics* **60:** (3) 341.

Matula M, Kypr J. 1999. Acad Sci Czech Republ, Inst Biophys, Kralovopolska 135, CZ-61265 Brno, Czech Republic. Nucleotide sequences flanking dinucleotide microsatellites in the human, mouse and *Drosophila* genomes. *J Biomol Struct Dyn* **17:** (2) 275.

McDonald JP, Rapic-Otrin V, Epstein JA, Broughton BC, Wang XY, Lehmann AR, Wolgemuth DJ, Woodgate R*. 1999. NIH/NICHHD, Sect DNA Replicat Repair & Mutagenesis, Bethesda, Md 20892, USA. Novel human and mouse homologs of *Saccharomyces cerevisiae* DNA polymerase $\eta$. *Genomics* **60:** (1) 20.

Nagasawa A, Kudoh J, Noda S, Mashima Y, Wright A, Oguchi Y, Shimizu N*. 1999. *Keio Univ, Sch Med, Dept Mol Biol, Shinjuku ku, 35 Shinanomachi, Tokyo 160 8582, Japan. Human and mouse ISLR (immunoglobulin superfamily containing leucine-rich repeat) genes: Genomic structure and tissue expression. *Genomics* **61:** (1) 37.

Rasmusson AG, Svensson AS, Knoop V, Grohmann L, Brennicke A. 1999. Lund Univ, Dept Plant Physiol, Box 117, SE-22100 Lund, Sweden. Homologues of yeast and bacterial rotenone-insensitive NADH dehydrogenases in higher eukaryotes: Two enzymes are present in potato mitochondria. *Plant J* **20:** (1) 79.

Sanchez M, Calzada A, Bueno A*. 1999. *Univ Salamanca, CSIC, Dept Microbiol & Genet Edificio Departamental, Ctr Invest Canc, ES-37007 Salamanca, Spain. Functionally homologous DNA replication genes in fission and budding yeast. *J Cell Sci* **112:** (14) 2381.

Sedlacek Z, Munstermann E, Dhorne-Pollet S, Otto C, Bock D, Schutz G, Poustka A*. 1999. *Deutsch Krebsforschungszentrum, Mol Genomanal, Neuenheimer Feld 280, DE-69120 Heidelberg, Germany. Human and mouse *XAP-5* and *XAP-5-like* (*X5L*) genes: Identification of an ancient functional retroposon differentially expressed in testis. *Genomics* **61:** (2) 125.

Sillje HHW, Takahashi K, Tanaka K, Van Houwe G, Nigg EA*. 1999. *Univ Geneva, Dept Mol Biol, 30 Quai Ernest-Ansermet, CH-1211 Geneva 4, Switzerland. Mammalian homologues of the plant *Tousled* gene code for cell-cycle-regulated kinases with maximal activities linked to ongoing DNA replication. *EMBO J* **18:** (20) 5691.

Sundstrom J, Carlsbecker A, Svensson ME, Svenson M, Johanson U, Theissen G, Engstrom P*. 1999. *Uppsala Univ, Evolutionary Biol Ctr, Dept Physiol Bot, Villavagen 6, SE-75236 Uppsala, Sweden. MADS-box genes active in developing pollen cones of Norway spruce (*Picea abies*) are homologous to the B-class floral homeotic genes in angiosperms. *Dev Genetics* **25:** (3) 253.

Van Dodeweerd AM, Hall CR, Bent EG, Johnson SJ, Bevan MW, Bancroft I*. 1999. *John Innes Ctr Plant Sci Res, Norwich Res Pk, Norwich NR4 7UH, England. Identification and analysis of homoeologous segments of the genomes of rice and *Arabidopsis thaliana*. *Genome* **42:** (5) 887.

Yamaguchi F, Yamaguchi K, Tokuda M, Brenner S. 1999. Inst Mol Sci, 2168 Shattuck Ave, Berkeley, Ca 94704, USA. Molecular cloning of *EDG-3* and *N-Shc* genes from the puffer fish, *Fugu rubripes*, and conservation of synteny with the human genome. *FEBS Lett* **459:** (1) 105.

### 6 Gene families and regulons

Ade J, Belzile F, Philippe H, Doutriaux MP*. 1999. *Univ Paris II, CNRS, URA 8618, Inst Plantes Biotechnol, BAT 630, FR-91405 Orsay, France. Four mismatch repair paralogues coexist in *Arabidopsis thaliana*: *AtMSH2, AtMSH3, AtMSH6-1* and *AtMSH6-2*. *Mol Gen Genet* **262:** (2) 239.

Baker RT, Wang XW, Wollatt E, Shite JA, Sutherland GR. 1999. Australian Natl Univ, John Curtin Sch Med Res, Div Mol Med, Ubiquitin Lab, GPO Box 334, Canberra, ACT 2601, Australia. Identification, functional characterization, and chromosomal localization of USP15, a novel human ubiquitin-specific protease related to the UNP oncoprotein, and a systematic nomenclature for human ubiquitin-specific proteases. *Genomics* **59:** (3) 264.

Conklin D, Lofton-Day CE, Haldeman RA, Ching A, Whitmore TE, Lok S, Jaspers S*. 1999. Zymogenet Inc, 1201 Eastlake Ave East, Seattle, Wa 98102, USA. Identification of INSL5, a new member of the insulin super family. *Genomics* **60:** (1) 50.

Delneri D, Gardner DCJ, Bruschi CV, Oliver SG*. 1999. *UMIST, Sch Biol Sci, 2.205 Stopford Bldg, Manchester M60 1QD, England. Disruption of seven hypothetical aryl alcohol dehydrogenase genes from *Saccharomyces cerevisiae* and construction of a multiple knock-out strain. *Yeast* **15:** (15) 1681.

Krupnik VE, Sharp JD, Jiang C, Robison K, Chickering TW, Amaravadi L, Brown DE, Guyot D, Mays G, Leiby K, Chang B, Duong T, Goodearl ADJ, Gearing DP, Sokol SY, McCarthy SA*. 1999. *Millennium Bio Therapeut Inc, 640 Mem Dr, Cambridge, Ma 02139, USA. Functional and structural diversity of the human Dickkopf gene family.

Copyright © 2000 John Wiley & Sons, Ltd.

*Gene* **238:** (2) 301.

Lee GC, Tang SJ*, Sun KH, Shaw JF. 1999. *Acad Sinica, Inst Bot, Taipei 11529, Taiwan. Analysis of the gene family encoding lipases in *Candida rugosa* by competitive reverse transcription-PCR. *Appl Environ Microbiol* **65:** (9) 3888.

Llorente B, Fairhead C, Dujon B. 1999. Univ Paris 06, Inst Pasteur, CNRS, URA 1300, UFR927, Dept Biotechnol, Unite Genet Mol Levures, FR-75724 Paris 15, France. Genetic redundancy and gene fusion in the genome of the baker's yeast *Saccharomyces cerevisiae*: Functional characterization of a three-member gene family involved in the thiamine biosynthetic pathway. *Mol Microbiol* **32:** (6) 1140.

Meissner RC, Jin HL, Cominelli E, Denekamp M, Fuertes A, Greco R, Kranz HD, Penfield S, Petroni K, Urzainqui A *et al*. 1999. c/o Bevan M, John Innes Ctr, Dept Mol Genet, Colney Lane, Norwich Res Pk, Norwich NR4 7UH, England. Function search in a large transcription factor gene family in *Arabidopsis*: Assessing the potential of reverse genetics to identify insertional mutations in R2R3 *MYB* genes. *Plant Cell* **11:** (10) 1827.

Nakashima H, Grahovac MJ, Mazzarella R, Fujiwara H, Kitchen JR, Threat TA, Ko MSH*. 1999. *NIH/NIA, Genet Lab, Dev Genom & Aging Sect, 333 Cassell Dr, Triad Suite 4000, Baltimore, Md 21224, USA. Two novel mouse genes-*Nubp2*, mapped to the *t*-complex on chromosome 17, and *Nubp1*, mapped to chromosome 16: Establish a new gene family of nucleotide-binding proteins in eukaryotes. *Genomics* **60:** (2) 152.

Sakumoto N, Mukai Y, Uchida K, Kouchi T, Kuwajima J, Nakagawa Y, Sugioka S, Yamamoto E, Furuyama T, Mizubuchi H, Ohsugi N, Sakuno T, Kikuchi K, Matsuoka I, Ogawa N, Kaneko Y, Harashima S*. 1999. *Osaka Univ, Grad Sch Engn, Dept Biotechnol, 2-1 Yamadaoka, Suita, Osaka 565-0871, Japan. A series of protein phosphatase gene disruptants in *Saccharomyces cerevisiae*. *Yeast* **15:** (15) 1669.

Shimozawa N, Imamura A, Zhang ZY, Suzuki Y, Orii T, Tsukamoto T, Osumi T, Fujiki Y, Wanders RJA, Besley G, Kondo N. 1999. Gifu Univ, Sch Med, Dept Paediat, 40 Tsukasa machi, Gifu 500 8076, Japan. Defective *PEX* gene products correlate with the protein import, biochemical abnormalities, and phenotypic heterogeneity in peroxisome biogenesis disorders. *J Med Genet* **36:** (10) 779.

Takahashi Y, Nakamura M. 1999. Osaka Univ, Grad Sch Sci, Dept Biol, 1-1- Machikaneyama, Toyonaka, Osaka 560 0043, Japan. Functional assignment of the ORF2-*iscS-iscU-iscA-hscB-hscA-fdx*-ORF3 gene cluster involved in the assembly of Fe-S clusters in *Escherichia coli*. *J Biochem Tokyo* **126:** (5) 917.

### 7 Pharmacogenomics

Afshari CA, Nuwaysir EF, Barrett JC*. 1999. *NIEHS, Mol Carcinogenesis Lab, POB 12233, Res Triangle Park, NC 27709, USA. Application of complementary DNA microarray technology to carcinogen identification, toxicology, and drug safety evaluation. *Cancer Res* **59:** (19) 4759.

Bubendorf L, Kononen J, Koivisto P, Schraml P, Moch H, Gasser TC, Willi N, Mihatsch MJ, Sauter G, Kallioniemi OP*. 1999. *NIH/Natl Human Genome Res Inst, Canc Genet Branch, 49 Convent Dr, Room 4A24, Bethesda, Md 20892, USA. Survey of gene amplifications during prostate cancer progression by high throughput fluorescence *in situ* hybridization on tissue microarrays. *Cancer Res* **59:** (4) 803.

Golub TR, Slonim DK, Tamayo P, Huard C, Gaasenbeek M, Mesirov JP, Coller H, Loh ML, Downing JR, Caligiuri MA Bloomfield CD, Lander ES. 1999. MIT, Whitehead Inst, Ctr Genome Res, 77 Massachusetts Ave, Cambridge, Ma 02139, USA. Molecular classification of cancer: Class discovery and class prediction by gene expression monitoring. *Science* **286:** (5439) 531.

Howell SB. 1999. Univ Calif San Diego, Dept Med, 9500 Gilman Dr, La Jolla, Ca 92093, USA. DNA microarrays for analysis of gene expression in prostate cancer. *Mol Urol* **3:** (3) 295.

Nuwaysir EF, Bittner M, Trent J, Barrett JC, Afshari CA. 1999. NIEHS, Mol Carcinogenesis Lab, 111 Alexander Dr, Res Triangle Park, NC 27709, USA. Microarrays and toxicology: The advent of toxicogenomics. *Mol Carcinog* **24:** (3) 153.

Sapolsky RJ, Hsie L, Berno A, Ghandour G, Mittmann M, Fan JB*. 1999. *Affymetrix Inc, 3380 Cent Expressway, Santa Clara, Ca 95051, USA. High-throughput polymorphism screening and genotyping with high-density oligonucleotide arrays. *Genet Anal-Biomol Eng* **14:** (5-6) 187.

Whitney LW, Becker KG, Tresser NJ, Caballero-Ramos CI, Munson PJ, Prabhu VV, Trent JM, McFarland HF, Biddison WE. 1999. NIH/NINCDS, Mol Immunol Sect, Neuroimmunol Branch, Bldg 10, Room 5B-16, 10 Ctr Dr, Bethesda, Md 20892, USA. Analysis of gene expression in multiple sclerosis lesions using cDNA microarrays. *Ann Neurol* **46:** (3) 425.

### 8 Large-scale mutagenesis programmes

Bianchi MM, Sartori G, Ven den Bol M, Kaniak A, Uccelletti D, Mazzoni C, Di Rago JP, Carignani G, Slonimski PP, Frontali L*. 1999. Univ Rome La Sapienza, Dept Cell & Develop Biol, Ple Aldo Moro 5, IT-00185 Rome, Italy. How to bring orphan genes into functional families. *Yeast* **15:** (6) 513.

Liu LX, Spoerke JM, Mulligan EL, Chen J, Reardon B, Westlund B, Sun L, Abel K, Armstrong B, Hardiman G, King J, MacCague L, Basson M, Clover R, Johnson CD. 1999. Cambria Biosci, Waltham, Ma 02454, USA. High-throughput isolation of *Caenorhabditis elegans* deletion mutants. *Genome Res* **9:** (9) 859.

Niedenthal R, Riles L, Guldener U, Klein S, Johnston M, Hegemann JH*. 1999. *Heinrich-Heine-Univ, Inst Mikrobiol, Universitatstr 1, Geb. 26.12.01.64, DE-40225 Dusseldorf, Germany. Systematic analysis of *S. cerevisiae* chromosome VIII genes. *Yeast* **15:** (16) 1775.

Redman RS, Ranson JC, Rodriguez RJ*. 1999. *USGS, Western Fisheries Res Ctr, Biiol Resources Div, 6505 NE 65th St, Seattle, Wa 98115, USA. Conversion of the pathogenic fungus *Colletotrichum magna* to a nonpathogenic, endophytic mutualist by gene disruption. *Mol Plant Microbe Interact* **12:** (11) 969.

Winzeler EA, Shoemaker DD, Astromoff A, Liang H, Anderson K, Andre B, Bangham R, Benito R, Boeke JD, Bussey H *et al*. 1999. c/o Davis RW, Stanford Univ, Sch Med, Dept Biochem, Stanford, Ca 94305, USA. Functional characterization of the *S. cerevisiae* genome by gene deletion and parallel analysis. *Science* **285:** (5429) 901.

### 9 Functional complementation

Foti DM, Welihinda A, Kaufman RJ, Lee AS*. 1999. *Univ Sthn Calif, Kenneth Norris Jr Comprehens Canc Ctr, Dept Biochem & Mol Biol, Sch Med, Los Angeles, Ca 90089, USA. Conservation and divergence of the yeast and mammalian unfolded protein response: Activation of specific mammalian endoplasmic reticulum stress element of the *grp*78/BiP promoter by yeast Hac1. *J Biol Chem* **274:** (43) 30402.

Galichet A, Belarbi A*. 1999. *Univ Reims, UFR Sci, Lab Microbiol

Copyright © 2000 John Wiley & Sons, Ltd.

Gen & Mol, POB 1039, FR-51687 Reims 2, France. Cloning of an α-glucosidase gene from *Thermococcus hydrothermalis* by functional complementation of a *Saccharomyces cerevisiae mal11* mutant strain. *FEBS Lett* **458:** (2) 188.

Pan ZQ, Chang CR*. 1999. *Univ Maryland, HJ Patterson Hall, Dept Cell Biol & Mol Genet, College Park, Md 20742, USA. Functional complementation of the *Schizosaccharomyces pombe wis1* mutant by *Arabidopsis MEK1* and non-catalytic enhancement by *CTR1*. *FEBS Lett* **459:** (3) 405.

Sriskanda V, Schwer B, Ho CK, Shuman S*. 1999. *Sloan Kettering Inst, Program Mol Biol, New York, NY 10021, USA. Mutational analysis of *Escherichia coli* DNA ligase identifies amino acids required for nick-ligation *in vitro* and for *in vivo* complementation of the growth of yeast cells deleted for CDC9 and LIG4. *Nucleic Acids Res* **27:** (20) 3953.

Symula DJ, Frazer KA, Ueda Y, Denefle P, Stevens ME, Wang ZE, Locksley R, Rubin EM*. 1999. *Lawrence Berkeley Lab, Genome Sci Dept, Berkeley, Ca 94720, USA. Functional screening of an asthma QTL in YAC transgenic mice. *Nat Genet* **23:** (2) 241.

Viola AM, Lodi T, Ferrero I. 1999. Univ Parma, Inst Genet, Area Parco Sci 11-A, IT-43100 Parma, Italy. A *Klaac* null mutant of *Kluyveromyces lactis* is complemented by a single copy of the *Saccharomyces cerevisiae AAC1* gene. *Curr Genet* **36:** (1-2) 29.

Wongwathanarat P, Michaelson LV, Carter AT, Lazarus CM, Griffiths G, Stobart AK, Archer DB, MacKenzie DA*. 1999. *AFRC Inst Food Res, Colney Lane, Norwich NR4 7UA, England. Two fatty acid Δ9-desaturase genes, *ole1* and *ole2*, from *Mortierella alpina* complement the yeast *ole1* mutation. *Microbiology* **145:** (10) 2939.

### 10 Transcriptomics

Alon U, Barkai N, Notterman DA, Gish K, Ybarra S, Mack D, Levine AJ*. 1999. *Rockefeller Univ, Presidents Off, 1230 York Ave, New York, 10021, USA. Broad patterns of gene expression revealed by clustering analysis of tumor and normal colon tissues probed by oligonucleotide arrays. *Proc Natl Acad Sci U S A* **96:** (12) 6745.

Amundson SA, Bittner M, Chen YD, Trent J, Meltzer P, Fornace AJ. 1999. NIH/NCI, 37 Convent Dr, Bethesda, Md 20892, USA. Fluorescent cDNA microarray hybridization reveals complexity and heterogeneity of cellular genotoxic stress responses. *Oncogene* **18:** (24) 3666.

Behr MA, Wilson MA, Gill WP, Salamon H, Schoolnik GK, Rane S, Small PM. 1999. McGill Univ, Ctr Hlth, Dept Med, Div Infect Dis, Montreal, Quebec, Canada H3G 1A4. Comparative genomics of BCG vaccines by whole-genome DNA microarray. *Science* **284:** (5419) 1520.

Cairney J, Xu NF, Pullman GS, Ciavatta VT, Johns B. 1999. Inst Paper Sci & Technol, Forest Biol Grp, 500 10th St NW, Atlanta, Ga 30318, USA. Natural and somatic embryo development in loblolly pine: Gene expression studies using differential display and DNA arrays. *Appl Biochem Biotechnol* **77-9:** 5.

Gelfand MS, Mironov AA, Jomantas J, Kozlov YI, Perumov DA. 1999. State Ctr Biotechnol, Dorozhny pr, RU-113545 Moscow 1, Russia. A conserved RNA structure element involved in the regulation of bacterial riboflavin synthesis genes. *Trends Genet* **15:** (11) 439.

Granjeaud S, Bertucci F, Jordan BR*. 1999. *CIML Luminy, Case 906, FR-13288 Marseille 9, France. Expression profiling: DNA arrays in many guises. *Bioessays* **21:** (9) 781.

Herrero P, Flores L, De la Cera T, Moreno F*. 1999. *Univ Oviedo, Inst Univ Biotechnol, Dept Bioquim & Biol Mol, ES-33006 Oviedo, Spain. Functional characterization of transcriptional regulatory elements in the upstream region of the yeast *GLK1* gene. *Biochem J* **343:** (2) 319.

Koch RA, Thiele DJ*. 1999. *Univ Michigan, Sch Med, Dept Biol Chem, 1301 Catherine Rd, Ann Arbor, Mi 48109, USA. Functional analysis of a homopolymeric (dA-dT) element that provides nucleosomal access to yeast and mammalian transcription factors. *J Biol Chem* **274:** (34) 23752.

Lelivelt MJ, Culbertson MR*. 1999. *Univ Wisconsin, Mol Biol Lab, Bock Labs 435A, 1525 Linden Dr, Madison, Wi 53706, USA. Yeast Upf proteins for RNA surveillance affect global expression of the yeast transcriptome. *Mol Cell Biol* **19:** (10) 6710.

Xiong LM, Ishitani M, Lee HJ, Zhu JK*. 1999. *Univ Arizona, Dept Plant Sci, Tucson, Az 85721, USA. Hos5: A negative regulator of osmotic stress-induced gene expression in *Arabidopsis thaliana*. *Plant J* **19:** (5) 569.

Zhang MQ. 1999. Cold Spring Harbor Lab, POB 100, 1 Bungtown Rd, Cold Spring Harbor, NY 11724, USA. Promoter analysis of co-regulated genes in the yeast genome. *Comput Chem* **23:** (3-4) 233.

### 11 Proteomics

Drobyshev AL, Zasedatelev AS, Yershov GM, Mirzabekov AD*. 1999. *Argonne Natl Lab, Biochip Technol Ctr, 9700 Sth Cass Ave, Argonne, Il 60439, USA. Massive parallel analysis of DNA-Hoechst 33258 binding specificity with a generic oligodeoxyribonucleotide microchip. *Nucleic Acids Res* **27:** (20) 4100.

Futcher B, Latter GI, Monardo P, McLaughlin CS, Garrels JI. 1999. Cold Spring Harbor Lab, POB 100, Cold Spring Harbor, NY 11724, USA. A sampling of the yeast proteome. *Mol Cell Biol* **19:** (11) 7357.

Gauss C, Kalkum M, Lowe M, Lehrach H, Klose J*. 1999. *Humboldt Univ, Inst Humangenet, Klin Rudolf Virchow, Augustenburger Pl 1, DE-13353 Berlin, Germany. Analysis of the mouse proteome. (I) - Brain proteins: Separation by two-dimensional electrophoresis and identification by mass spectrometry and genetic variation. *Electrophoresis* **20:** (3) 575.

Gygi SP, Rist B, Gerber SA, Turecek F, Gelb MH, Aebersold R*. 1999. *Univ Washington, Dept Mol Biotechnol, Box 357730, Seattle, Wa 98195, USA. Quantitative analysis of complex protein mixtures using isotope-coded affinity tags. *Nat Biotechnol* **17:** (10) 994.

Jensen PK, Pasa-Tolic L, Anderson GA, Horner JA, Lipton MS, Bruce JE, Smith RD*. 1999. *Pacific NW Lab, Environm Mol Sci Lab, POB 999, Richland, Wa 99352, USA. Probing proteomes using capillary isoelectric focusing-electrospray ionization Fourier transform ion cyclotron resonance mass spectrometry. *Anal Chem* **71:** (11) 2076.

Rigaut G, Shevchenko A, Rutz B, Wilm M, Mann W, Seraphin B*. 1999. *European Mol Biol Lab, Meyerhofstr 1, DE-69117 Heidelberg, Germany. A generic protein method for protein complex characterization and proteome exploration. *Nat Biotechnol* **17:** (10) 1030.

Walhout AJM, Sordella R, Lu X, Hartley JL, Temple GF, Brasch NTM, Vidal M. 2000. Harvard Univ, Med Sch, Dana Farber Canc Inst, Dept Genet, Boston, Ma 02115, USA. Protein interaction mapping in *C. elegans* using proteins involved in vulval development. *Science* **287:** (5450) 116.

### 13 Metabolomics

Hong SP, Piper MD, Sinclair DA, Dawes IW*. 1999. *Univ New Sth

Copyright © 2000 John Wiley & Sons, Ltd.

Wales, Sch Biochem & Mol Genet, Sydney, NSW 2052, Australia. Control of expression of one-carbon metabolism genes of *Saccharomyces cerevisiae* is mediated by a tetrahydrofolate-responsive protein binding to a glycine regulatory region including a core 5'-CTTCTT-3' motif. *J Biol Chem* **274:** (15) 10523.

Keasling JD. 1999. Univ Calif, Dept Chem Engn, Berkeley, Ca 94720, USA. Gene-expression tools for the metabolic engineering of bacteria (Review). *Trends Biotechnol* **17:** (11) 452.

Kwast KE, Burke PV, Staahl BT, Poyton RO*. 1999. *Univ Colorado, Dept Mol Cellular & Dev Biol, Boulder, Co 80309, USA. Oxygen sensing in yeast: Evidence for the involvement of the respiratory chain in regulating the transcription of a subset of hypoxic genes. *Proc Natl Acad Sci U S A* **96:** (10) 5446.

Liu ZC, Butow RA*. 1999. *Univ Texas, SW Med Ctr, Dept Mol Biol, 5323 Harry Hines Blvd, Dallas, Tx 75235, USA. A transcriptional switch in the expression of yeast tricarboxylic acid cycle genes in response to a reduction or loss of respiratory function. *Mol Cell Biol* **19:** (10) 6720.

Tadi D, Hasan RN, Bussereau F, Boy-Marcotte E*, Jacquet M. 1999. *Univ Paris-Sud, UMR C8621, Inst Genet & Microbiol, Lab Inform Genet & Dev, Bat 400, FR-91405 Orsay, France. Selection of genes repressed by cAMP that are induced by nutritional limitation in *Saccharomyces cerevisiae*. *Yeast* **15:** (16) 1733.

### 14 Genomic approaches to development

Gao FB, Brenman JE, Jan LY, Jan YN*. 1999. *Univ Calif, Howard Hughes Med Inst, Dept Physiol, San Francisco, Ca 94143, USA. Genes regulating dendritic outgrowth, branching, and routing in *Drosophila*. *Gene Dev* **13:** (19) 2549.

Siegfried KR, Eshed Y, Baum SF, Otsuga D, Drews GN, Bowman JL*. 1999. *Univ Calif, Plant Biol Sect, Davis, Ca 95616, USA. Members of the *YABBY* gene family specify abaxial cell fate in *Arabidopsis*. *Development* **126:** (18) 4117.

Yan YT, Gritsman K, Ding JX, Burdine RD, Corrales JD, Price SM, Talbot WS, Schier AF*, Shen MM. 1999. *NYU, Sch Med, Skirball Inst Biomol Med, Dev Genet Program, Dept Cell Biol, New York, NY 10016, USA. Conserved requirement for *EGF-CFC* genes in vertebrate left-right axis formation. *Gene Dev* **13:** (19) 2527.

Zhao DH, Yang M, Solava J, Ma H*. 1999. *Penn State Univ, Life Sci Consortium, Wartik Lab 504, Dept Biol, University Park, Pa 16802, USA. The *ASK1* gene regulates development and interacts with the *UFO* gene to control floral organ identity in *Arabidopsis*. *Dev Genetics* **25:** (3) 209.

### 15 Technological advances

Ahrendt SA, Halachmi S, Chow JT, Wu L, Halachmi N, Yang SC, Wehage S, Jen J, Sidransky D*. 1999. *Johns Hopkins Univ, Sch Med, Dept Otolaryngol Head & Neck Surg, 720 Rutland Ave, Baltimore, Md 21287, USA. Rapid p53 sequence analysis in primary lung cancer using an oligonucleotide probe array. *Proc Natl Acad Sci U S A* **96:** (13) 7382.

Baldwin D, Crane V, Rice D. 1999. Pioneer Hi-Bred Int Inc, Trait & Technol Dev, Dis Resistance Grp, 7300 NW 62nd Ave, Johnston, Ia 50131, USA. A comparison of gel-based, nylon filter and microarray techniques to detect differential RNA expression in plants. *Curr Opin Plant Biol* **2:** (2) 96.

Bardea A, Patolsky F, Dagan A, Willner I*. 1999. *Hebrew Univ, Inst Chem, IL-91904 Jerusalem, Israel. Sensing and amplification of oligonucleotide-DNA interactions by means of impedence spectroscopy: A route to a Tay-Sachs sensor. *Chem Commun* (1) 21.

Bardea A, Dagan A, Willner I*. 1999. *Hebrew Univ Jerusalem, Inst Chem, IL-91904 Jerusalem, Israel. Amplified electronic transduction of oligonucleotide interactions: Novel routes for Tay-Sachs biosensors. *Anal Chim Acta* **385:** (1-3) 33.

Bazin H, Livache T. 1999. CIS Bio Int DIVT Res & New Technol, BP 175, FR-30203 Bagnols-sur-Ceze, France. Peptide- and biotin-oligonucleotide-pyrrole conjugates for electrochemical addressing on silicon chip. *Nucleosides Nucleotides* **18:** (6-7) 1309.

Beier M, Hoheisel JD. 1999. Deutsch Krebsforschungszentrum, Neuenheimer Feld 506, DE-69120 Heidelberg, Germany. New developments in light-controlled synthesis of DNA-arrays. *Nucleosides Nucleotides* **18:** (6-7) 1301.

Bell PJL, Davies IW, Attfield PV. 1999. Macquarie Univ, Sch Biol Sci, Sydney, NSW 2109, Australia. Facilitating functional analysis of the *Saccharomyces cerevisiae* genome using an EGFP-based promoter library and flow cytometry. *Yeast* **15:** (16) 1747.

Berggren C, Stalhandske P, Brundell J, Johansson G*. 1999. *Lund Univ, Dept Anal Chem, PO Box 124, SE-22100 Lund, Sweden. A feasibility study of a capacitive biosensor for direct detection of DNA hybridization. *Electroanalysis* **11:** (3) 156.

Bertucci F, Bernard K, Loriod B, Chang YC, Granjeaud S, Bimbaum D, Nguyen C, Peck K, Jordan BR*. 1999. *CIML Luminy, FR-13288 Marseille 9, France. Sensitivity issues in DNA array-based expression measurements and performance of nylon microarrays for small samples. *Hum Mol Genet* **8:** (9) 1715.

Brockman JM, Frutos AG, Corn RM*. 1999. *Univ Wisconsin, Dept Chem, 1101 Univ Ave, Madison, Wi 53706, USA. A multistep chemical modification procedure to create DNA arrays on gold surfaces for the study of protein-DNA interactions with surface plasmon resonance imaging. *J Am Chem Soc* **121:** (35) 8044.

Budach W, Abel AP, Bruno AE, Neuschafer D. 1999. Novartis Pharma AG, DMPK, CH-4002 Basel, Switzerland. Planar waveguides as high performance sensing platforms for fluorescence-based multiplexed oligonucleotide hybridization assays. *Anal Chem* **71:** (16) 3347.

Certa U, Hochstrasser R, Langen H, Buess M, Moroni C. 1999. F Hoffmann La Roche & Co Ltd, Grenzacherstr 127, CH-4070 Basel, Switzerland. Biosensors in biomedical research: Development and applications of gene chips. *Chimia* **53:** (3) 57.

Che DP, Bao P, Li R, Daywalt M, Ruffalo T, Shi JF, Prokhorova A, Lublinsky A, Lermer N, Muller UR. 1999. Vysis Inc, Downers Grove, IL, USA. A novel surface, attachment chemistry, and CCD-based multicolor imaging system for analysis of genomic DNA arrays. *Scanning* **21:** (2) 63.

Chou HP, Spence C, Scherer A, Quake S*. 1999. *Calif Inst Technol, Dept Appl Phys, MS 128-95, Pasadena, Ca 91125, USA. A microfabricated device for sizing and sorting DNA molecules. *Proc Natl Acad Sci U S A* **96:** (1) 11.

Christel LA, Petersen K, McMillan W, Northrup MA. 1999. CEPHEID, 1190 Borregas Ave, Sunnyvale, Ca 94089, USA. Rapid, automated nucleic acid probe assays using silicon microstructures for nucleic acid concentration. *J Biomech Eng* **121:** (1) 22.

Courtney BC, Smith MM, Henchal EA. 1999. US Army, Med Res Inst Infect Dis, Diagnost Syst Div, 1425 Porter St, Fort Detrick, Md 21702, USA. Development of internal controls for probe-based nucleic acid diagnostic assays. *Anal Biochem* **270:** (2) 249.

Copyright © 2000 John Wiley & Sons, Ltd. 

Durfee T, Draper O, Zupan J, Conklin DS, Zambryski PC. 1999. Univ Calif, Dept Plant & Microbial Biol, 111 Koshland Hall, Berkeley, Ca 94720, USA. New tools for protein linkage mapping and general two-hybrid screening. *Yeast* **15:** (16) 1761.

Ehrlich DJ, Matsudaira P. 1999. Whitehead Inst Biomed Res, 9 Cambridge Ctr, Cambridge, Ma 02142, USA. Microfluidic devices for DNA analysis. *Trends Biotechnol* **17:** (8) 315.

Fan ZH, Mangru S, Granzow R, Heaney P, Ho W, Dong QP, Kumar R. 1999. Sarnoff Corp, 201 Washington Rd, Princeton, NJ 08543, USA. Dynamic DNA hybridization on a chip using paramagnetic beads. *Anal Chem* **71:** (21) 4851.

Fang XH, Liu XJ, Schuster S, Tan WH*. 1999. *Univ Florida, Dept Chem, Gainesville, Fl 32601, USA. Designing a novel molecular beacon for surface-immobilized DNA hybridization studies. *J Am Chem Soc* **121:** (12) 2921.

Favorov AV, Livshits MA, Mirzabekov AD. 1999. Russian Acad Sci, Engelhardt Inst Mol Biol, RU-117984 Moscow, Russia. DNA hybridization of oligonucleotide microchip. Correlations and screening for errors. *Mol Biol-Engl Tr* **33:** (3) 374.

Fidanza JA, McGall GH. 1999. Affymetrix Inc, 3380 Cent Expressway, Santa Clara, Ca 95051, USA. High-density nucleoside analog probe arrays for enhanced hybridization. *Nucleosides Nucleotides* **18:** (6-7) 1293.

Garnier F, Korri-Youssoufi, Srivastava P, Mandrand B, Delair T. 1999. CNRS, Lab Mat Mol, 9 rue Henri Dumant, FR-94305 Thiais, France. Toward intelligent polymers: DNA sensors based on oligonucleotide-functionalized polypyrroles. *Synthet Metal* **100:** (1) 89.

Gerhold D, Rushmore T, Caskey CT. 1999. Merck & Co Inc, Dept Human Genet, West Point, Pa 19486, USA. DNA chips: Promising toys have become powerful tools. *Trends Biochem Sci* **24:** (5) 168.

Gerry NP, Witowski NE, Day J, Hammer RP, Barany G, Barany F*. 1999. *Cornell Univ, Joan & Sandford I Weill Med Coll, Dept Microbiol, Hearst Microbiol Res Ctr, New York, NY 10021, USA. Universal DNA microarray method for multiplex detection of low abundance point mutations. *J Mol Biol* **292:** (2) 251.

Gilles PN, Wu DJ*, Foster CB, Dillon PJ, Chanock SJ. 1999. *Nanogen Inc, 10398 Pacific Ctr Ct, San Diego, Ca 92121, USA. Single nucleotide polymorphic discrimination by an electronic dot blot assay on semiconductor microchips. *Nat Biotechnol* **17:** (4) 365.

Hacia JG, Fan JB, Ryder O, Jin L, Edgemon K, Ghandour G, Mayer RA, Sun B, Hsie L, Robbins CM, Brody LC, Wang D, Lander ES, Lipshutz R, Fodor SPA, Collins FS*. 1999. *Natl Human Genome Res Inst, Bldg 49-3A14, Bethesda, Md 20892, USA. Determination of ancestral alleles for human single-nucleotide polymorphisms using high-density oligonucleotide arrays. *Nat Genet* **22:** (2) 164.

Henn W. 1999. Univ Saarland, Inst Human Genet, DE-6650 Homburg, Germany. Genetic screening with the DNA chip: A new Pandora's box? *J Med Ethics* **25:** (2) 200.

Kehoe DM, Villand P, Somerville S*. 1999. *Carnegie Inst Washington, Dept Plant Biol, 290 Panama St, Stanford, Ca 94305, USA. Technical focus: DNA microarrays for studies of higher plants and other photosynthetic organisms. *Trends Plant Sci* **4:** (1) 38.

Kuipers OP, De Jong A, Holsappel S, Bron S, Kok J, Hamoen LW. 1999. Univ Groningen, Dept Genet, Groningen Biomol Sci & Biotechnol Inst, POB 14, NL-9750 AA Haren, The Netherlands. DNA-microarrays and food-biotechnology. *Antonie van Leeuwenhoek* **76:** (1) 353.

Liu SR, Shi YN, Ja WW, Mathies RA*. 1999. *Univ Calif, Dept Chem, Berkeley, Ca 94720, USA. Optimization of high-speed DNA sequencing on microfabricated capillary electrophoresis channels. *Anal Chem* **71:** (3) 566.

Lueking A, Horn M, Eickhoff H, Bussow K, Lehrach H, Walter G. 1999. Max Planck Inst Mol Genet, Ihnestr 73, DE-14195 Berlin, Germany. Protein microarrays for gene expression and antibody screening. *Anal Biochem* **270:** (1) 103.

Lunt DH, Hutchinson WF, Carvalho GR. 1999. Univ Hull, Dept Sci Biol, Mol Ecol & Fisheries Genet Lab, Hull HU6 7RX, England. An efficient method for PCR-based isolation of microsatellite arrays (PIMA). *Mol Ecol* **8:** (5) 891.

Mahadevappa M, Warrington JA. 1999. Affymetrix Inc, High Throughput Screening, 3380 Cent Expressway, Santa Clara, Ca 95051, USA. A high-density probe array sample preparation method using 10 to 100-fold fewer cells. *Nat Biotechnol* **17:** (11) 1134.

Marrazza G, Chianella I, Mascini M*. 1999. *Sez Chim Anal, Dipt Sanita Pubbl Epidemiol Chim Anal Ambient, via G Capponi 9, IT-50121 Firenze, Italy. Disposable DNA electrochemical sensor for hybridization detection. *Biosens Bioelectron* **14:** (1) 43.

Mikulits W, Dolznig H, Hofbauer R, Mullner EW*. 1999. *Univ Vienna, Inst Mol Biol, Vienna Bioctr, Dr Bohr Gasse 9, AU-1030 Vienna, Austria. Reverse strand priming: A versatile cDNA radiolabeling method for differential hybridization on nucleic acid arrays. *Biotechniques* **26:** (5) 846.

Mir KU, Southern EM. 1999. Univ Oxford, Dept Biochem, Sth Parks Rd, Oxford OX1 3QU, England. Determining the influence of structure on hybridization using oligonucleotide arrays. *Nat Biotechnol* **17:** (8) 788.

Moch H, Schraml P, Bubendorf L, Mirlacher M, Kononen J, Gasser T, Mihatsch MJ, Kallioniemi OP, Sauter G. 1999. Univ Basel, Inst Pathol, Schonbeinstr 40, CH-4003 Basel, Switzerland. High-throughput tissue microarray analysis to evaluate genes uncovered by cDNA microarray screening in renal cell carcinoma. *Am J Pathol* **154:** (4) 981.

.Morozov VN, Morozova TY. 1999. NYU, Dept Chem, WM Keck Fdn Lab, Biomol Imaging, New York, NY 10003, USA. Electrospray deposition as a method for mass fabrication of mono- and multicomponent microarrays of biological and biologically active substances. *Anal Chem* **71:** (15) 3110.

Niemeyer CM, Blohm D. 1999. Univ Bremen, FB2-UFT Biotechnol & Molek Genet, Leobener Str, DE-28359 Bremen, Germany. DNA microarrays. *Angew Chem Int Ed* **38:** (19) 2865.

Nilsson P, Larsson A, Lundeberg J, Uhlen M, Nygren PA. 1999. Royal Inst Technol, KTH, Dept Biotechnol, SE-10044 Stockholm, Sweden. Mutational scanning of PCR products by subtractive oligonucleotide hybridization analysis. *Biotechniques* **26:** (2) 308.

Ostroff RM, Hopkins D, Haeberli AB, Baouchi W, Polisky B. 1999. Biostar Inc, 6655 Lookout Rd, Boulder, Co 80301, USA. Thin film biosensor for rapid visual detection of nucleic acid targets. *Clin Chem* **45:** (9) 1659.

Pena J, Ricke SC, Shermer CL, Gibbs T, Pillai SD*. 1999. *Texas A&M Univ, Res Ctr, Environm Sci Program, El Paso, Tx 79927, USA. A gene amplification-hybridization sensor based methodology to rapidly screen aerosol samples for specific bacterial gene sequences. *J Environ Sci Health A* **34:** (3) 529.

Pollack JR, Perou CM, Alizadeh AA, Eisen MB, Pergamenschikov A, Williams CF, Jeffrey SS, Botstein D, Brown PO*. 1999. *Stanford Univ, Howard Hughes Med Inst, Med Ctr, Stanford, Ca 94305, USA. Genome-wide analysis of DNA copy-number changes using cDNA microarrays. *Nat Genet* **23:** (1) 41.

Rogers J. 1999. Wellcome Trust Res Labs, Sanger Ctr, Genome Cam-

Copyright © 2000 John Wiley & Sons, Ltd. 

pus, Cambridge CB10 1SA, England. Sequencing: Gels and genomes. *Science* **286:** (5439) 429.

Rogers YH, Jiang-Baucom P, Huang ZJ, Bogdanov V, Anderson S, Boyce-Jacino MT*. 1999. *Orchid Biocomp Inc, Alpha Ctr, Johns Hopkins Bayview Res Campus, 5210 Eastern Ave, Baltimore, Md 21224, USA. Immobilization of oligonucleotides onto a glass support *via* disulfide bonds: A method for preparation of DNA microarrays. *Anal Biochem* **266:** (1) 23.

Roget A, Livache T*. 1999. *CEA Grenoble, DRFMC, CIS Bio Int, 17 rue Martyrs, FR-38054 Grenoble 9, France. *In situ* synthesis and copolymerization of oligonucleotides on conducting polymers. *Mikrochim Acta* **131:** (1-2) 3.

Rowe CA, Tender LM, Feldstein MJ, Golden JP, Scruggs SB, MacCraith BD, Cras JJ, Ligler FS*. 1999. *US Navy, Res Lab, Ctr Biomol Sci & Engn, Code 6900, Washington, DC 20375, USA. Array biosensor for simultaneous identification of bacterial, viral, and protein analytes. *Anal Chem* **71:** (17) 3846.

Sachadyn P, Kur J. 1999. Gdansk Tech Univ, Dept Microbiol, ul Narutowicza 11-12, PL-80952 Gdansk, Poland. Reducing the number of microlocations in oligonucleotide microchip matrices by the application of degenerate oligonucleotides. *J Theor Biol* **197:** (3) 393.

Sawata S, Kai E, Ikebukuro K, Iida T, Honda T, Karube I*. 1999. *Univ Tokyo, Res Ctr Adv Sci & Technol, 4-6-1 Komaba, Meguro-ku, Tokyo 153-8904, Japan. Application of peptide nucleic acid to the direct detection of deoxyribonucleic acid amplified by polymerase chain reaction. *Biosens Bioelectron* **14:** (4) 397.

Scampavia LD, Hodder PS, Lahdesmaki I, Ruzicka J. 1999. Univ Washington, Dept Chem, Box 351700, Seattle, Wa 98195, USA. Automation of functional assays by flow injection fluorescence microscopy. *Trends Biotechnol* **17:** (11) 443.

Singh-Gasson S, Green RD, Yue YJ, Nelson C, Blattner F, Sussman MR*, Cerrina F. 1999. *Univ Wisconsin, Ctr Biotechnol, Madison, Wi 53706, USA. Maskless fabrication of light-directed oligonucleotide microarrays using a digital micromirror array. *Nat Biotechnol* **17:** (10) 974.

Sojka B, Piunno PAE, Wust CC, Krull UJ*. 1999. *Univ Toronto, Dept Chem, Chem Sensors Grp, 3359 Mississauga Rd Nth, Mississauga, Ontario, Canada L5L 1C6. Evaluating the quality of oligonucleotides that are immobilized on glass supports for biosensor development. *Anal Chim Acta* **395:** (3) 273.

Speulman E, Metz PJL, Van Arkel G, Hekkert PTL, Stiekema WJ, Pereira A*. 1999. *Ctr Plant Breeding & Reprod Res, Dept Biol Mol, POB 16, NL-6700 AA Wageningen, The Netherlands. A two-component enhancer-inhibitor transposon mutagenesis system for functional analysis of the *Arabidopsis* genome. *Plant Cell* **11:** (10) 1853.

Sturniolo T, Bono E, Ding JY, Raddrizzani L, Tuereci O, Sahin U, Braxenthaler M, Gallazzi F, Protti MP, Sinigaglia F, Hammer J*. 1999. *Roche Milano Ric, IT-20132 Milan, Italy. Generation of tissue-specific and promiscuous HLA ligand databases using DNA microarrays and virtual HLA class II matrices. *Nat Biotechnol* **17:** (6) 555.

Tang K, Fud J, Julien D, Braun A, Cantor CR, Koster H. 1999. Sequenom Inc, 11555 Sorrento Valley Rd, San Diego, Ca 92121, USA. Chip-based genotyping by mass spectrometry. *Proc Natl Acad Sci U S A* **96:** (18) 10016.

Thompson M, Furtado LM. 1999. Univ Toronto, Dept Chem, 80 St George St, Toronto, Ontario, Canada K5S 3H6. High density oligonucleotide and DNA probe arrays for the analysis of target DNA. *Analyst* **124:** (8) 1133.

Tissier AF, Marillonnet S, Klimyuk V, Patel K, Torres MA, Murphy G, Jones JDG*. 1999. *John Innes Ctr Plant Sci Res, Colney Lane, Norwich NR4 7UH, England. Multiple independent defective suppressor-mutator transposon insertions in *Arabidopsis*: A tool for functional genomics. *Plant Cell* **11:** (10) 1841.

Trenkle T, Welsh J, McClelland M*. 1999. *Sidney Kimmel Canc Ctr, 10835 Altman Row, San Diego, Ca 92121, USA. Differential display probes for cDNA arrays. *Biotechniques* **27:** (3) 554.

Vente A, Korn B, Zehetner G, Poustka A, Lehrach H. 1999. German Human Genome Project Berlin, Resource Ctr, Heubnerweg 6, DE-14059 Berlin, Germany. Distribution and early development of microarray technology in Europe (Letter). *Nat Genet* **22:** (1) 22.

Vidal M, Legrain P. 1999. MGH, Canc Ctr, Charlestown, Ma 02129, USA. Yeast forward and reverse 'n'-hybrid systems. *Nucl Acid Res* **27:** (4) 919.

Vo Dinh T, Alarie JP, Isola N, Landis D, Wintenberg AL, Ericson MN. 1999. Oak Ridge Natl Lab, Oak Ridge, Tn 37831, USA. DNA biochip using a phototransistor integrated circuit. *Anal Chem* **71:** (2) 358.

Wang J. 1999. State Univ, Dept Chem & Biochem, POB 30001, Las Cruces, NM 88003, USA. Towards genoelectronics: Electrochemical biosensing of DNA hybridization. *Chem-Eur J* **5:** (6) 1681.

Wang YX, Rea T, Bian JH, Gray S, Sun Y*. 1999. *Parke Davis Pharmaceut Res, Dept Mol Biol, 2800 Plymouth Rd, Ann Arbor, Mi 48105, USA. Identification of the genes responsive to etoposide-induced apoptosis: Application of DNA chip technology. *FEBS Lett* **445:** (2-3) 269.

Watson SJ, Akil H. 1999. 205 Zina Pitcher Pl, Ann Arbor, Mi 48109, USA. Gene chips and arrays revealed: A primer on their power and their uses. *Biol Psychiatry* **45:** (5) 533.

Xu DK, Ma LR, Liu YQ, Jiang ZH, Liu ZH. 1999. Chinese Natl Biomed Anal Ctr, Inst Radiat Med, 27 Taiping Rd, CN-100850 Beijing, Peoples Rep China. Development of chemiluminescent biosensing of nucleic acids based on oligonucleotide-immobilized gold surfaces. *Analyst* **12:** (4) 533.

Zen JM, Chang MR, Ilangovan G. 1999. Natl Chunghsing Univ, Dept Chem, Taichung 402, Taiwan. Simultaneous determination of guanine and adenine contents in DNA, RNA and synthetic oligonucleotides using a chemically modified electrode. *Analyst* **124:** (5) 679.

### 16 Bioinformatics

De Pinto B, Malladi SB, Altamura N*. 1999. *Univ Bari, CNR, Ctr Studio Mitocondri & Metab Energet, via Amendola 165-A, IT-70126 Bari, Italy. MitBASE pilot: A database on nuclear genes involved in mitochondrial biogenesis and its regulation in *Saccharomyces cerevisiae*. *Nucl Acid Res* **27:** (1) 147.

Galperin MY, Koonin EV. 1999. NIH/Natl Lib Med, Natl Ctr Biotechnol Informat, Bethesda, Md 20894, USA. Functional genomics and enzyme evolution: Homologous and analogous enzymes encoded in microbial genomes. *Genetica* **106:** (1-2) 159.

Hodges PE, McKee AHZ, Davis BP, Payne WE, Garrels JI*. 1999. *Proteome Inc, Cummings Ctr 100, Suite 435M, Beverly, Ma 01915, USA. The yeast proteome database (YPD): A model for the organization and presentation of genome-wide functional data. *Nucleic Acids Res* **27:** (1) 69.

Hokamp K, Wolfe K. 1999. Univ Dublin, Trinity Coll, Dept Genet, Dublin 2, Rep Ireland. What's new in the library? What's new in GenBank? Let PubCrawler tell you. *Trends Genet* **15:** (11) 471.

Copyright © 2000 John Wiley & Sons, Ltd. 
